# Retraction Note: *In vitro* and *in vivo* responses of macrophages to magnesium-doped titanium

**DOI:** 10.1038/s41598-021-88664-z

**Published:** 2021-04-20

**Authors:** Bin Li, Huiliang Cao, Yaochao Zhao, Mengqi Cheng, Hui Qin, Tao Cheng, Yan Hu, Xianlong Zhang, Xuanyong Liu

**Affiliations:** 1 Department of Orthopedics, Shanghai Sixth People’s Hospital, Shanghai Jiao Tong University, Shanghai, 200233 China; 2 State Key Laboratory of High Performance Ceramics and Superfine Microstructure, Shanghai Institute of Ceramics, Chinese Academy of Sciences, Shanghai, 200050 China

Retraction of: *Scientific Reports* 10.1038/srep42707, published online 15 February 2017

The Editors have retracted this Article. After publication, concerns were raised that some panels within Figures 6 and 7 contain repeated features.

In addition, it was found that some of the original data related to Figure 6 are missing.

The Editors therefore no longer have confidence in the integrity of the data presented.

Xuanyong Liu and Huiliang Cao agree with the retraction. Bin Li and Xianlong Zhang disagree with the retraction. Yaochao Zhao, Mengqi Cheng, Hui Qin, Tao Cheng and Yan Hu did not respond to the correspondence about the retraction.

